# Glycol vapor pathogen disinfection: a defensive technology for emerging biothreats

**DOI:** 10.3389/fmicb.2026.1843269

**Published:** 2026-05-29

**Authors:** Sarah Siddiqui, Jaspreet Pannu

**Affiliations:** 1 Center for Health Security, Bloomberg School of Public Health, Johns Hopkins University, Baltimore, MD, United States; 2 Department of Environmental Health and Engineering, Bloomberg School of Public Health, Johns Hopkins University, Baltimore, MD, United States

**Keywords:** aerosolized anti-microbials, air-borne transmission, environmental inactivation, non-pharmaceutical interventions, pandemic preparedness

## Abstract

Airborne transmission of respiratory pathogens has substantial societal and economic burdens during seasonal epidemics and pandemics. Therefore, there is a need for built environment interventions that prevent pathogen spread indoors. Glycol vapors were historically investigated as air disinfectants and have recently regained attention. Yet their mechanism of action, effectiveness across different environments, and safety remains incompletely defined. Here we describe historical and modern evidence on glycol vapor-mediated pathogen inactivation in the air. Experimental studies have shown rapid reductions in airborne microbial viability under controlled conditions. The data suggest that antimicrobial activity arises from humidity-dependent partitioning of glycol vapors into respiratory droplets, altering droplet chemistry and destabilizing pathogen structures. Indoor environment parameters including humidity, temperature, ventilation, and droplet lifetime were found to strongly influence efficacy. Translating findings from chamber studies into real-world indoor occupied spaces remains challenging. Here, we discuss how sensor-informed modeling and machine-learning approaches could support control of glycol vapor systems by integrating environmental measurements. Existing occupational exposure data from glycol-based theatrical fogs provides useful safety information, however further dedicated exposure studies are still needed. Overall, glycol vapors represent a plausible non-pharmaceutical airborne infection control strategy whose value depends on defining environmental operating conditions and safety limits for continuous deployment.

## 1 Introduction

Respiratory viruses cause severe societal and economic burdens particularly during the winter months when increased time spent indoors promotes transmission (Shaw Stewart, 2016; Putri et al., 2018; Neumann and Kawaoka, 2022). These costs can escalate dramatically during outbreaks or pandemics (Cutler and Summers, 2020). Public health initiatives including mask-wearing and lockdowns can reduce transmission (Flaxman et al., 2020; Haug et al., 2020). But the effectiveness of these measures depends on public compliance, which can be challenging to achieve, especially for diseases with low fatality rates. Consequently, additional strategies that enhance ventilation, air filtration, or environmental disinfection could help reduce respiratory virus transmission.

Propylene glycol compound was “generally recognized as safe” (GRAS) and is used in a wide variety of products including food, cosmetics, and pharmaceuticals (Agency for Toxic Substances and Disease Registry (ATSDR), 1997; European Medicines Agency, 2013; US Food Drug Administration, 2021). Propylene glycol (PG) and triethylene glycol (TEG) are hygroscopic molecules that have been shown to have potent antimicrobial and antiviral effects in vapor form (Duggan et al., 2024). Glycol vapors were first explored for air disinfection in the mid-20th century (Robertson et al., 1941a; Puck et al., 1943) and have seen renewed interest in recent years for indoor air disinfection (Duggan et al., 2024). The mechanisms by which glycol vapors inactivate microbes are not yet fully understood, though they are thought to involve membrane permeabilization, protein disruption, and interference with viral replication complexes (Styles et al., 2023; Duggan et al., 2024).

Glycol vapors are cheap and could be rapidly deployed, though potential safety concerns must be evaluated. Established approaches such as HEPA filtration have a strong evidence base for reducing airborne pathogens and are widely used in buildings. However, not all buildings have sufficient airflow to support powerful filters. Far-UVC light is highly effective at inactivating pathogens and is likely to be safe for human exposure because its narrow wavelength window (~200–222 nm) damages microbes and does not significantly penetrate skin or eyes (Buonanno et al., 2017). However, additional research is needed to optimize its deployment and reduce the costs of far-UVC light sources such as krypton chloride excimer lamps. Compared to these methods, glycol vapors offer a potentially inexpensive and scalable approach to air disinfection as they do not require major modifications to building ventilation systems. The use of HEPA and high MERV filters in HVAC systems places higher energy demands on fans to maintain airflow and results in higher operational costs (Zaatari et al., 2014). In many existing buildings, HVAC systems cannot accommodate high-efficiency filtration without equipment upgrades such as ductwork modification and fan replacement (Board on Chemical Sciences and Technology Board on Life Sciences Division on Earth and Life Studies Environmental Health Matters Initiative and National Academies of Sciences Engineering and Medicine, 2023). Far-UVC systems may offer greater benefits for similar costs compared to HEPA filters (Matysik et al., 2025) but require multiple distributed emitters to achieve whole-room disinfection (Cummings et al., 2025). Glycol vapors may offer a complementary strategy where ventilation or filtration upgrades are not feasible. However, unlike HEPA filtration, there is limited evidence on the effectiveness of glycol vapors in real-world environments. Further research is needed to characterize their performance and safety. As a result, while filtration remains the most established intervention and far-UVC is an emerging option, glycol vapors may warrant further investigation as a potentially scalable approach in constrained settings.

## 2 Disinfection activity of glycol vapors across pathogen classes

Early-to-mid 20th-century studies showed glycol vapors reduced airborne microbe viability, though using approaches that predate modern aerobiology standards. More recent studies using controlled aerosol chamber systems have reproduced these findings with modern viability assays, providing stronger experimental support for glycol-mediated microbe inactivation. In the sections below, we discuss the evidence across different pathogen classes.

### 2.1 Enveloped viruses

The evidence for glycol vapor-mediated air disinfection is strongest for enveloped respiratory viruses. Early aerosol experiments and modern chamber experiments report substantial reductions in airborne infectivity of enveloped viruses following short exposures to glycol vapors. The first direct evidence that glycol vapors could suppress airborne transmission of influenza virus comes from animal studies (Robertson et al., 1941b). In mice survival studies using morbidity and mortality as measures of glycol efficacy, mice exposed to aerosolized influenza virus survived when PG vapor was present in the air compared to control groups (Robertson et al., 1941b). Similar protective effects were observed in large room experiments where glycol vapor prevented mortality in exposed animals at levels comparable to ultraviolet irradiation (Henle et al., 1942). Complementary pediatric hospital ward studies showed that PG and TEG vapor reduced transmission of respiratory infections including viral infections during respiratory season (Harris and Stokes, 1945).

Recent controlled aerosol chamber studies have reproduced these findings. PG vapor was shown to inactivate both aerosolized SARS-CoV-2 (Hirama et al., 2023; Styles et al., 2023) and Influenza A virus in a dose-dependent manner (Styles et al., 2023). Exposure to Influenza A virus in the presence of PG vapor also resulted in survival of mice (Styles et al., 2023). Aerosolized TEG has also demonstrated measurable reductions in influenza virus viability on surfaces, with reported log-scale decreases in infectivity over time (Rudnick et al., 2009). Additionally, *in vitro* experiments using pseudotyped viral systems show that PG has broad virucidal activity against several enveloped viruses including NL63 and 229E seasonal coronaviruses, SARS-CoV2 variants, middle eastern respiratory syndrome coronavirus (MERS) and Ebola (Styles et al., 2023). Taken together, historical and modern studies provide consistent evidence that glycol vapors can inactivate enveloped respiratory viruses.

### 2.2 Non-enveloped viruses

Evidence for glycol vapor-mediated inactivation of non-enveloped viruses is more limited and some experimental settings have shown reduced susceptibility compared with enveloped respiratory viruses. In controlled chamber studies, exposure to TEG vapor has been reported to reduce airborne infectivity of the non-enveloped MS2 bacteriophage by approximately 2–3 log (Desai et al., 2023, 2026). Similarly, *in vitro* experiments showed that PG reduced infectivity of rotavirus, though effective inactivation typically requires elevated glycol concentrations or higher temperatures relative to enveloped virus models (Styles et al., 2023). These reports suggest that glycol vapors inactivate non-enveloped viruses, but the magnitude and kinetics of inactivation are generally less pronounced than those observed for enveloped respiratory viruses.

### 2.3 Bacteria

The antimicrobial activity of glycol vapors against bacteria is the earliest and most extensively documented application of glycol vapor air disinfection. Laboratory experiments reported rapid declines in aerosolized hemolytic streptococci viability following short exposures to PG vapor (Robertson et al., 1941a; Puck et al., 1943). Animal transmission models further supported these findings. PG vapor reduced airborne transmission of streptococcal infection between animals in chamber studies (Henle et al., 1942). Pediatric hospital ward studies conducted during respiratory disease season reported reductions in bacterial counts and decreased incidence of respiratory infections when PG or TEG vapors were maintained in enclosed spaces (Harris and Stokes, 1945). Reductions in recoverable circulating bacteria were consistently observed when sufficient vapor concentrations were achieved.

Glycol vapors were evaluated against a broad range of bacterial species representing both gram-positive and gram-negative organisms. Tested organisms included Streptococcus species (including hemolytic streptococci and pneumococci), Staphylococcus species, gram-negative bacteria such as Escherichia coli, Serratia marcescens, and multiple Salmonella species including Salmonella gallinarum and Salmonella pullorum (Robertson et al., 1941a; Puck et al., 1943; Moore, 1947; Braymen and Soncer, 1970; Pelleu et al., 1974). Recent studies have reproduced these findings using chamber systems. TEG vapor was found to reduce viability of many different gram-positive and gram-negative bacterial species including Mycobacterium species (Desai et al., 2026). This reinforces the breadth of antimicrobial activity observed in earlier work. Taken together, these studies provide consistent evidence that glycol vapors reduce airborne bacterial viability under experimental conditions.

### 2.4 Fungi

In addition to bacteria and viruses, glycol vapors have also been evaluated on fungal spores. Initial chamber studies with aerosolized *Penicillium notatum* spores showed that TEG vapor effectively inactivated fungal spores (Mellody and Bigg, 1946). More recently, an office room study showed TEG vapor was effective in reducing airborne fungi and fungi on surfaces (Truyols-Vives et al., 2024). Taken together, these data suggested that glycol vapors can reduce airborne fungal viability under experimental conditions, although evidence is limited and largely restricted to a small number of species. The extent to which these findings extend to clinically relevant fungi remains unclear.

## 3 Mechanism of glycol vapor-mediated pathogen inactivation

**Figure 1:**
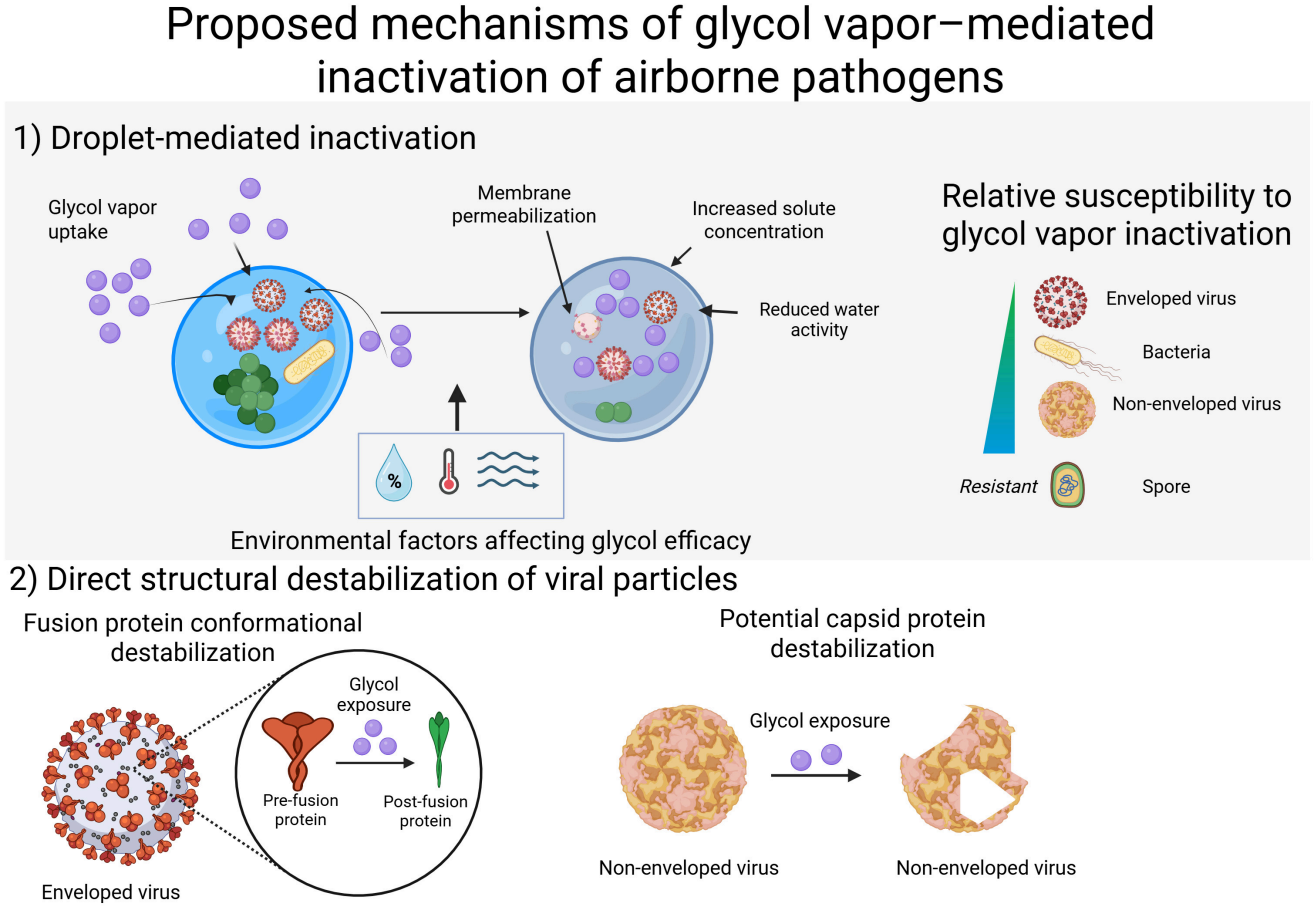
Proposed mechanisms of glycol vapor-mediated inactivation of airborne pathogens. Glycol vapors such as propylene glycol (PG) and triethylene glycol (TEG) can inactivate airborne pathogens through multiple physicochemical mechanisms. In droplet-mediated inactivation **(top)**, glycol vapors enter pathogen-containing respiratory droplets through condensation or dissolution, increasing local glycol concentrations in droplets. This alters droplet chemistry by increasing solute concentration and reducing water activity, which can lead to membrane permeabilization and microbial inactivation. Environmental factors such as relative humidity, temperature, and air exchange influence glycol uptake and antimicrobial efficacy. Glycols may also directly destabilize viral particles **(bottom)**. For enveloped viruses, glycol exposure may induce conformational destabilization of viral fusion proteins, promoting premature transition from pre-fusion to post-fusion states that render virions non-infectious. For non-enveloped viruses, glycols may destabilize capsid proteins, potentially leading to loss of capsid integrity. Susceptibility to glycol vapor inactivation varies across pathogen classes, with enveloped viruses generally more sensitive than bacteria, non-enveloped viruses, and spores. Created in BioRender. Siddiqui, S. (2026) https://BioRender.com/8l64uzk.

Studies have demonstrated that glycol vapors, particularly TEG and PG, can inactivate airborne microorganisms through a combination of physical and biochemical mechanisms (Figure 1). Early studies (Puck et al., 1943; Puck, 1947a,b) and subsequent investigations (Rubiano et al., 2020; Styles et al., 2023) indicate that a central process involves glycol vapor uptake into pathogen-containing respiratory droplets by condensation (direct transition of vapor onto the droplet surface) or partitioning (dissolution of vapor-phase glycol into the aqueous droplet). This results in high local glycol concentrations in the respiratory droplets that inactivate microbes by membrane permeabilization.

In addition to condensation-driven and surface-mediated effects, emerging mechanistic hypotheses suggest that glycol vapors may inactivate viruses through disruption of protein structure and conformational stability (Styles et al., 2023). These mechanisms vary across pathogen classes and environmental conditions. For enveloped viruses such as influenza viruses and coronaviruses, glycols may act on metastable viral surface proteins, promoting premature conformational transitions that render virions non-infectious. This proposed mechanism is consistent with the known structural fragility of viral fusion proteins, which must balance environmental stability with the ability to undergo membrane fusion upon host entry (White et al., 2008). For non-enveloped viruses, which possess more structurally stable capsids, higher glycol concentrations, longer exposure durations, or elevated temperatures may be required to induce comparable inactivation (Styles et al., 2023). Glycols would potentially disrupt capsid protein conformation resulting in virus inactivation (Styles et al., 2023). At sufficiently high local concentrations, some glycols may also exhibit weak detergent-like behavior, contributing to membrane disruption in enveloped viruses (Hirama et al., 2023).

## 4 Environmental factors affecting efficacy

Glycol vapor efficacy depends on environmental conditions such as temperature, humidity, filtration, and ventilation rates. Among these factors, relative humidity (RH) appears to play a central role. Early experiments demonstrated that the relationship between RH and antimicrobial efficacy depends on the compound's hygroscopicity (ability of a substance to attract, absorb, and retain water molecules from the surrounding environment). Experiments with hygroscopic disinfectants such as glycol vapors showed that the inactivation of aerosolized *Serratia marcescens* occurred at low relative humidity (25% RH) rather than at high humidity (80% RH) (Kethley et al., 1956). Among the tested compounds, TEG showed the highest efficacy at low humidity, followed by PG and then EG. The authors proposed that this difference arises from how RH alters the composition of respiratory droplets. For hygroscopic compounds such as glycols, higher RH promotes additional water uptake, diluting the glycol concentration within the droplet and reducing biocidal effectiveness (Kethley et al., 1956).

Although these early studies suggest a clear inverse relationship between RH and glycol efficacy, subsequent work indicates that this relationship is more complex and depends on additional environmental parameters. It was reported that the bactericidal activity of glycol vapors varied across humidity conditions in ways that could not be explained solely by RH, suggesting that other factors like droplet size and aerosol residence time also influence performance (Puck et al., 1943). A recent study examining TEG under simulated office conditions also demonstrated that environmental and building parameters strongly influence antiviral efficacy (Sultan et al., 2024). Using MS2 bacteriophage as a viral surrogate, TEG efficacy was found to decline with the increase in temperature from 22.0 to 25.0 °C with optimal temperature being either 22.0 or 23.5 °C (Sultan et al., 2024). TEG efficacy also peaked at 55 % RH, increased with higher recirculation rates but decreased with increasing ventilation rates and higher efficiency filters (Sultan et al., 2024). These findings highlight that glycol-mediated inactivation is governed not only by chemical concentration but also by indoor environmental conditions. Predicting real-world performance and guiding deployment strategies for glycol-based air disinfection would require defining these environmental conditions.

## 5 Safety and deployment constraints

Safety data for glycol vapors air disinfection are limited, but decades of occupational health research on theatrical fogs provide some useful evidence regarding exposure ranges and health effects. Theatrical fogs consist of glycol and water mixtures aerosolized using high-temperature heating elements to produce dense, visible fogs (Varughese et al., 2005) at much higher concentrations than those used for pathogen disinfection. Across multiple studies, average personal inhalable aerosol concentrations during theatrical use are 0.49 mg/m3, with short-term peak concentrations occasionally reaching several tens of mg/m3 (Teschke et al., 2005). Importantly, measured exposures did not exceed established occupational limits, including the ACGIH and WCB 8 h TWA limit of 10 mg/m3 for glycol mists, nor ANSI-recommended short-term peak limits (Teschke et al., 2005).

Health studies indicate that glycol-based fogs, at personal inhalable concentrations of 0.49 mg/m3, can cause acute nasal and throat irritation with drying-related symptoms (Varughese et al., 2005). These studies found that lung function was significantly lower among those working closest to the fog source, and chronic work-related wheezing and chest tightness were significantly associated with increased cumulative exposure to glycol fogs over the preceding 2 years (Teschke et al., 2005; Varughese et al., 2005).

Proposed air disinfection applications will disperse glycol vapors at substantially lower concentrations than those used for theatrical fog, ideally at concentrations that do not generate visible aerosols. For example, U.S. EPA chamber studies of a TEG-based antimicrobial vapor using a model bacteriophage, achieved significant reductions in aerosolized viral viability at average concentrations of approximately 1.2–1.5 mg/m3 (Desai et al., 2023). This concentration is comparable to background exposures measured near fog machines but are well below documented peak occupational exposures and established limits [ANSI E1.5-2009 (R2014)]. These differences suggest that disinfection-oriented applications may present lower risks than theatrical fog use.

An additional safety consideration is the potential formation of glycol degradation products under real-world operating conditions. Studies of heated glycol systems have documented formation of carbonyl compounds including formaldehyde and acetaldehyde from high-temperature applications like e-cigarettes, though glycol vapor disinfection would involve lower concentrations dispersed throughout indoor air (Jaegers et al., 2021; Høisæter et al., 2024). Byproduct formation should be evaluated under realistic operating conditions to ensure degradation products do not pose unintended health risks.

However, existing safety data remains indirect. Available studies reflect theatrical fog exposures or e-cigarette use rather than the conditions relevant to pathogen disinfection. Pathogen disinfection would involve continuous exposure in occupied buildings and use in settings that include vulnerable populations such as asthmatic and immunocompromised individuals. The key unanswered question is therefore not whether glycol vapors cause acute toxicity at low concentrations, but whether long-term routine exposure is safe under real-world use conditions. Dedicated studies evaluating continuous glycol vapor exposure, potential byproduct formation, and glycol sensitive populations will be necessary to establish confidence for routine indoor use.

## 6 AI-enabled optimization, sensing, and evaluation frameworks

No studies have applied artificial intelligence (AI) or machine learning (ML) approaches to glycol vapor disinfection systems. It is unclear how glycols would best be deployed in real-world indoor environments. Historical glycol vapor studies have been performed in unventilated chambers under controlled environmental conditions (Duggan et al., 2024). This is not representative of many indoor conditions where air is circulated and filtered by HVAC systems. Therefore, there is uncertainty in how chamber-derived efficacy data translates to real-world indoor environments. Modeling and ML methods capable of integrating microbial inactivation kinetics with indoor air dynamics could address this challenge. AI and ML methods are increasingly used to model indoor air quality and environmental systems (Wei et al., 2019; Latoń et al., 2025). Similarly, AI/ML methods could be used to optimize glycol vapor disinfection in real-world settings.

Development of predictive models that estimate pathogen inactivation as a function of environmental and building parameters should be prioritized. Such models could integrate variables including glycol vapor concentration, temperature, relative humidity, ventilation rate, filtration efficiency, and room geometry. ML models have previously been applied to predict indoor air pollutants such as CO_2_, particulate matter (PM_2_.5 and PM10), and volatile organic compounds from environmental and sensor data (Bakht et al., 2022; Wei et al., 2022). Similar approaches could be extended to glycol vapor systems by incorporating experimentally derived pathogen inactivation rates into predictive models that estimate disinfection performance under different building conditions.

AI may also play an important role in sensing and adaptive control of glycol vapor systems. Practical deployment would likely require environmental monitoring measuring variables such as temperature, humidity, occupancy, and glycol vapor levels. Machine learning models can infer indoor environmental conditions and forecast air quality dynamics from sensor data and weather inputs (Mottahedin et al., 2024). These models could be used to estimate real-time pathogen inactivation potential and identify deviations from intended operating conditions. Therefore, these approaches could provide a framework for translating laboratory evidence on glycol vapor disinfection into predictive models that guide safe and effective deployment in real-world indoor environments. These applications remain conceptual and represent a potential direction for future research.

## 7 Discussion

Glycol vapors represent a promising but underexplored strategy for reducing airborne pathogen transmission in indoor environments. Historical and modern studies demonstrate antimicrobial activity against multiple pathogen classes but the environmental conditions and mechanisms governing this activity remain incompletely defined. The most pressing knowledge gap concerns identifying the specific indoor conditions under which glycol vapor antimicrobial effects are sufficiently robust to provide meaningful public health benefits. Current evidence supports the role for glycol partitioning into respiratory droplets and subsequent physicochemical disruption, yet the relative contributions of different inactivation pathways remain unresolved. These uncertainties are especially important for resistant pathogens like non-enveloped viruses and spores, where evidence is limited.

Translating glycol vapor disinfection into practice will require defining the environmental and safety boundaries within which it can operate effectively. Future work should prioritize (1) animal and/or real-world efficacy studies (2) safety studies assessing long-term exposure during continuous indoor use (3) systematic evaluation (4) mechanistic studies. Integrating these data with modeling approaches capable of predicting efficacy under realistic building conditions will be critical for translating laboratory findings into practical deployment strategies. Answering these questions will determine whether glycol vapor systems can be safely and effectively used for indoor infection control and deployed during pandemics.
